# Improving accuracy in percutaneous lung puncture using thoracic respiratory synchronization and laser angle guidance

**DOI:** 10.3389/fmedt.2025.1659231

**Published:** 2025-09-19

**Authors:** Qingjie Yang, Qingtian Li, Shenghua Lv, Linhui Lan, Mingyang Wang, Kaibao Han

**Affiliations:** Department of Thoracic Surgery, Xiamen Humanity Hospital, Fujian Medical University, Xiamen, China

**Keywords:** percutaneous lung puncture, guider, laser, respiratory activity, pulmonary nodules

## Abstract

**Objective:**

To explore the feasibility of enhancing the accuracy of percutaneous lung puncture through matching the respiratory activity of the thorax and performing puncture under the guidance of a laser angle guider.

**Methods:**

A retrospective case-control study was adopted. Collected data of patients with pulmonary nodules undergoing puncture. They were categorized into the conventional puncture group (Con group), the laser guidance group (Laser group), and the thoracic respiratory activity matching group (Ram group) based on whether the puncture was guided by the laser angle guider and the application of the thorax respiratory activity matching technique.

**Results:**

277 patients were included: 96 in the Con group, 93 in the Laser group, and 88 in the Ram group. There were no statistically significant differences in the puncture purpose, age, gender, BMI, maximum diameter of pulmonary nodules, location of pulmonary nodules, and distance from the skin at the puncture point among the three groups (*P* > 0.05). Nevertheless, key outcomes showed the Ram group had better results than the Laser group, which were better than the Con group: the rate of reaching the predetermined position on the first puncture (67.05% vs. 37.63% vs. 23.96%), number of CT scans (3.66 ± 1.06 vs. 4.09 ± 1.05 vs. 4.50 ± 1.08 times), and procedure time (23.05 ± 13.89 vs. 28.83 ± 13.78 vs. 35.14 ± 14.20 min) (all *P* < 0.05). Complication rates were sequentially lower (7.95% vs. 16.13% vs. 26.04%; *P* = 0.146).

**Conclusion:**

Puncture by matching the thoracic respiratory activity and under the guidance of a laser angle guider can effectively improve the accuracy of percutaneous lung puncture, reduce complications. Furthermore, the procedure is straightforward, warranting further evaluation in larger, prospective studies.

**Clinical trial registration:**

Chinese Clinical Trial Registry (ChiCTR), identifier (ChiCTR2300069384).

## Introduction

Percutaneous lung puncture under CT (Computed Tomography) guidance is a critical procedure in thoracic oncology and respiratory medicine. Precise percutaneous lung puncture technology is a prerequisite for conducting diagnosis and treatment procedures such as lung tumor biopsy, ablation, and radioactive particle implantation. Nevertheless, in clinical practice, percutaneous puncture of smaller pulmonary nodules remains challenging. Frequently, repeated adjustments of the puncture needle's angle are necessary to reach a satisfactory position. This also raises the probability of complications like pneumothorax. Relevant reports indicate that the incidence of pneumothorax ranges from 13.0% to 38.0% (1–7). The respiratory movement of patients is one of the significant reasons for the difficulty in accurately puncturing smaller lung lesions (8). Currently, some robots applicable for percutaneous lung puncture are undergoing clinical trials (8, 9–11), mainly by employing magnetic navigation devices or respiratory gating techniques to overcome the influence of respiratory movement on percutaneous lung puncture. However, these devices are very costly and thus have not been widely utilized in clinical practice. Another crucial factor affecting the accuracy of percutaneous lung puncture is whether the operator can puncture the puncture needle to the target position at the pre-designed angle. This influencing factor can be overcome by auxiliary tools such as angle measuring instruments (12–14).

Our center has implemented percutaneous lung puncture cryoablation for early-stage lung cancer and pulmonary metastases. Since most lung lesions are smaller than 2 cm, there is a high requirement for the accuracy of percutaneous lung puncture. To enhance the puncture accuracy and reduce puncture complications, we initially designed a laser angle guider to guide the puncture (patent number: 202321187082.6) (15), making the puncture needle insertion direction more precise. Subsequently, we devised a thorax activity calibration plate to counteract the influence of respiratory movement on the puncture (patent number: 202321315967.X) (16). Eventually, the accuracy of percutaneous lung puncture was elevated by matching the thorax respiratory activity and combined with laser angle guidance. We conducted this retrospective study on the data of patients who underwent percutaneous lung puncture in our hospital.

## Materials & methods

### Ethics statement

This study was approved by the Medical Ethics Committee of Xiamen Humanity Hospital (NO. HAXM-EMC-20230103-001-01). All procedures performed in this study were in accordance with the ethical standards of the institutional and national research committee and with the Helsinki Declaration (as revised in 2013). Given its retrospective design, the ethics committee waived the requirement for informed consent from each patient. However, we still specifically obtained written informed consent from the patients involved in the pictures attached to this article.

### Data collection

The data of patients who underwent percutaneous lung puncture for lung lesion biopsy, ablation, and preoperative localization at Xiamen Humanity Hospital from January 2022 to September 2023 were gathered. As we started to employ the laser angle guider to guide percutaneous lung puncture in August 2022 and adopted the approach of matching the respiratory activity of the thorax in combination with laser angle guidance for puncture in March 2023, patients from January 2022 to July 2022 all received percutaneous lung puncture by the conventional method and were designated as the conventional puncture group (Con group). Patients from August 2022 to February 2023 who underwent percutaneous lung puncture under the guidance of the laser angle were classified as the laser guidance group (Laser group). Patients from March 2023 to September 2023 who underwent percutaneous lung puncture under the technology of matching thoracic respiratory activity combined with laser angle guidance were set as the thoracic respiratory activity matching group (Ram group).

Inclusion criteria for all patients: ① Patients undergoing percutaneous lung puncture biopsy for lung lesions, percutaneous lung puncture ablation, or preoperative percutaneous puncture positioning needle localization for pulmonary nodules. ② The maximum diameter of the lung lesion was no more than 2 cm. Exclusion criteria: ① Patients unable to cooperate during the percutaneous lung puncture operation. ② Patients with multiple pulmonary nodules undergoing simultaneous percutaneous puncture positioning or multiple lesions undergoing simultaneous puncture biopsy. ③ Patients with coagulation dysfunctions. ④ Patients with severe emphysema, bullae, or other high-risk factors at the lung puncture site that were prone to cause pneumothorax after puncture.

A total of 277 patients were enrolled, including 96 in the Con group, 93 in the Laser group, and 88 in the Ram group. To minimize operator-related bias, all patients were from the same medical team and all puncture procedures were performed by the same senior attending physician.

### Percutaneous lung puncture

#### Thoracic activity calibration plate

The Thoracic Activity Calibration Plate is an “L”-shaped device made of polyamide (PA) through 3D printing. It consists of two rectangular plates, each with dimensions of 25 cm × 20 cm × 0.5 cm (length × width × thickness). These two parts are connected by a rotating shaft at the bottom edge, allowing for the adjustment of the folding angle. On the top edge of the vertical part, there are specially designed dual—scale markings. The upper part is composed of dots with a diameter of 1 mm and a spacing of 2 mm, while the lower part consists of linear scales with a width of 1 mm and a spacing of 2 mm. During CT scanning, these scales can be clearly seen, enabling the identification of the positions corresponding to the scales. The device is also equipped with a positioning buckle and a lead—indicator strip. The positioning buckle contains an integrated linear laser emitter. The laser emitter is a common linear laser emitter that emits a straight red light, and there are no specific requirements for its precision. The lead—indicator strip is a silicone strip with dimensions of 3 cm × 1 cm, within which there is a lead wire measuring 0.1 cm × 3 cm. This enables the strip to be visualized during CT scanning.

**Figure 1:**
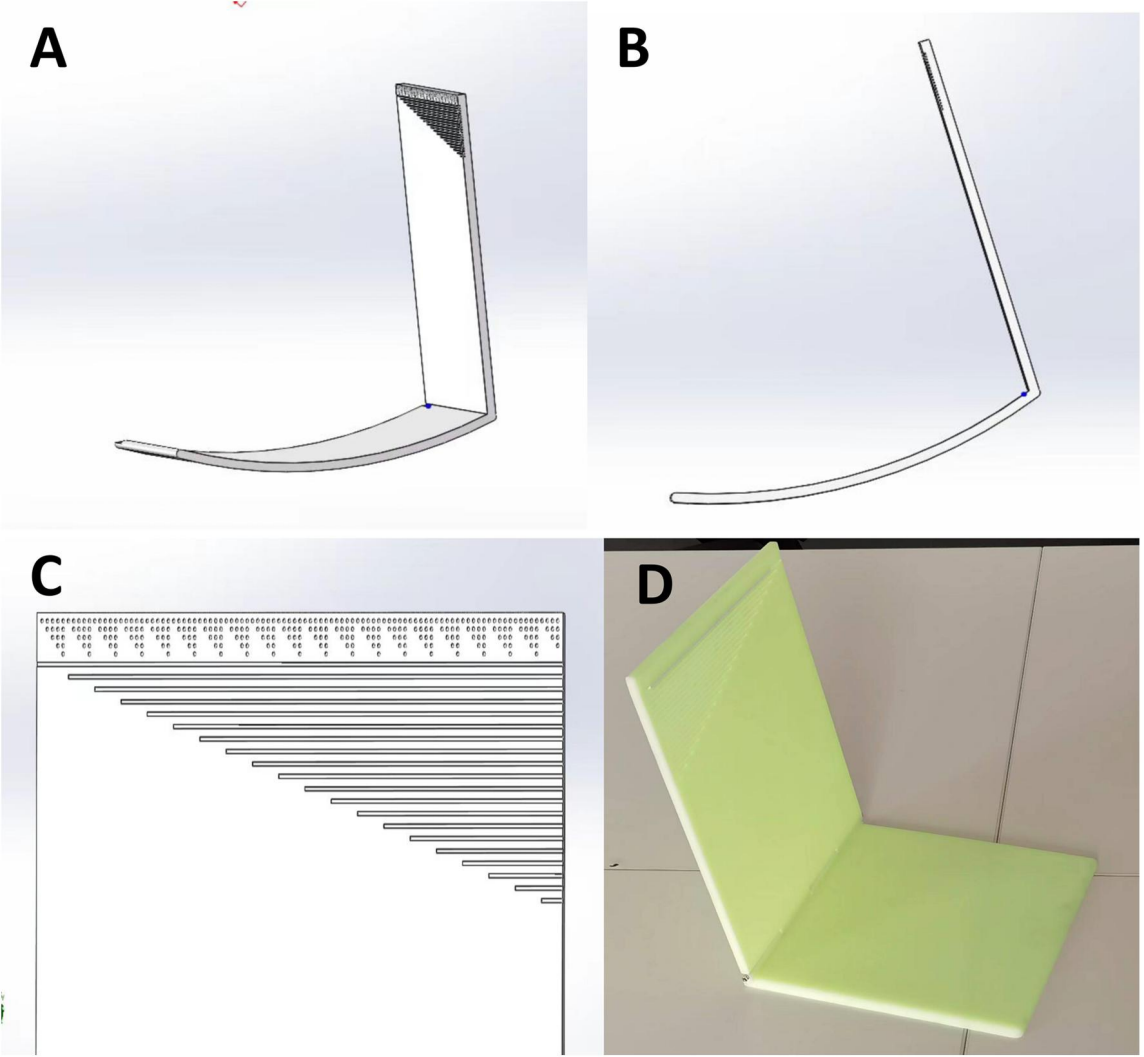
Thoracic activity calibration plate. **(A,B)** 3D pictures of thoracic activity calibration plate; **(C)** The scale of the plate; **(D)** The thoracic activity calibration plate printed by the 3D printer.

Its working principle is as follows: The “L”-shaped plate is placed on the CT bed. The indicator band is horizontally adhered to the patient's chest skin and moves along with the respiratory movement of the thorax. Consequently, the scale on the “L”-shaped plate indicated by the indicator band varies with the respiratory movement. During CT scan positioning, the scale on the “L”-shaped plate indicated by the indicator band is determined. When marking the skin puncture point and holding the puncture needle for the puncture, the patient adjusts the breathing amplitude to ensure that the scale on the “L”-shaped plate indicated by the indicator band is the same as that during CT scan positioning. This enables the relative position between the lung and the body surface during the puncture to be as consistent as possible with that during CT scan positioning, minimizing the impact of respiratory movement on the puncture. As it did not contact the sterile operative area, surface cleaning with 75% ethanol was performed after each procedure. See Figure 1.

#### Laser angle guider (LAG)

The Laser Angle Guider (LAG) consists of a peripheral frame and a laser angle meter. The peripheral frame is fabricated from 304 stainless steel and acrylic plates. It is externally furnished with a puncture needle fixator and internally utilized to accommodate the laser angle meter. The laser angle meter, a commercially available product, can display horizontal and vertical angles and emit cross positioning lasers.

**Figure 2:**
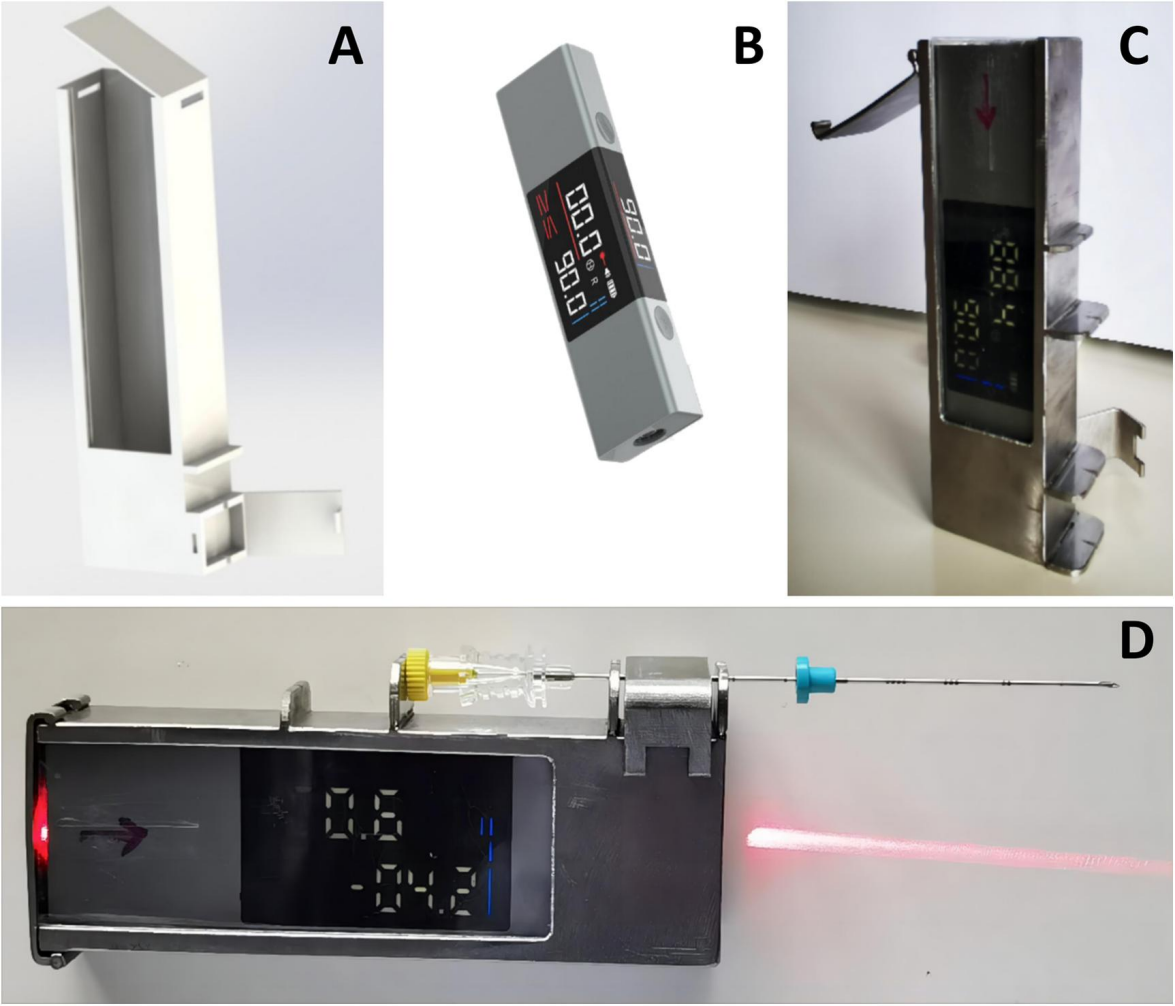
Laser angle guider. **(A)** 3D Picture of the peripheral frame of laser angle guider; **(B)** Laser angle meter; **(C)** The peripheral frame made of 304 stainless steel and acrylic plates, the laser angle meter was installed within the peripheral frame; **(D)** The puncture needle was installed on the laser angle guider.

Its working principle is as follows: The electronic angle meter measures the angle of the puncture needle insertion, making the puncture needle insertion angle consistent with the preset one. Meanwhile, the horizontal and vertical angle display of the laser angle meter and the laser projection can three-dimensionally guide the puncture. See Figure 2.

The peripheral frame of LAG is sterilized by ethylene oxide. The laser emitter, which is completely enclosed within the peripheral frame, does not require separate disinfection. During use, the operator opens the lid of the peripheral frame, and an assistant places the laser emitter inside the peripheral frame. Subsequently, the operator closes the lid to prevent direct contact between the laser emitter and the sterile area.

#### Percutaneous lung puncture under the technology of matching thoracic respiratory activity combined with laser angle guidance

**Figure 3:**
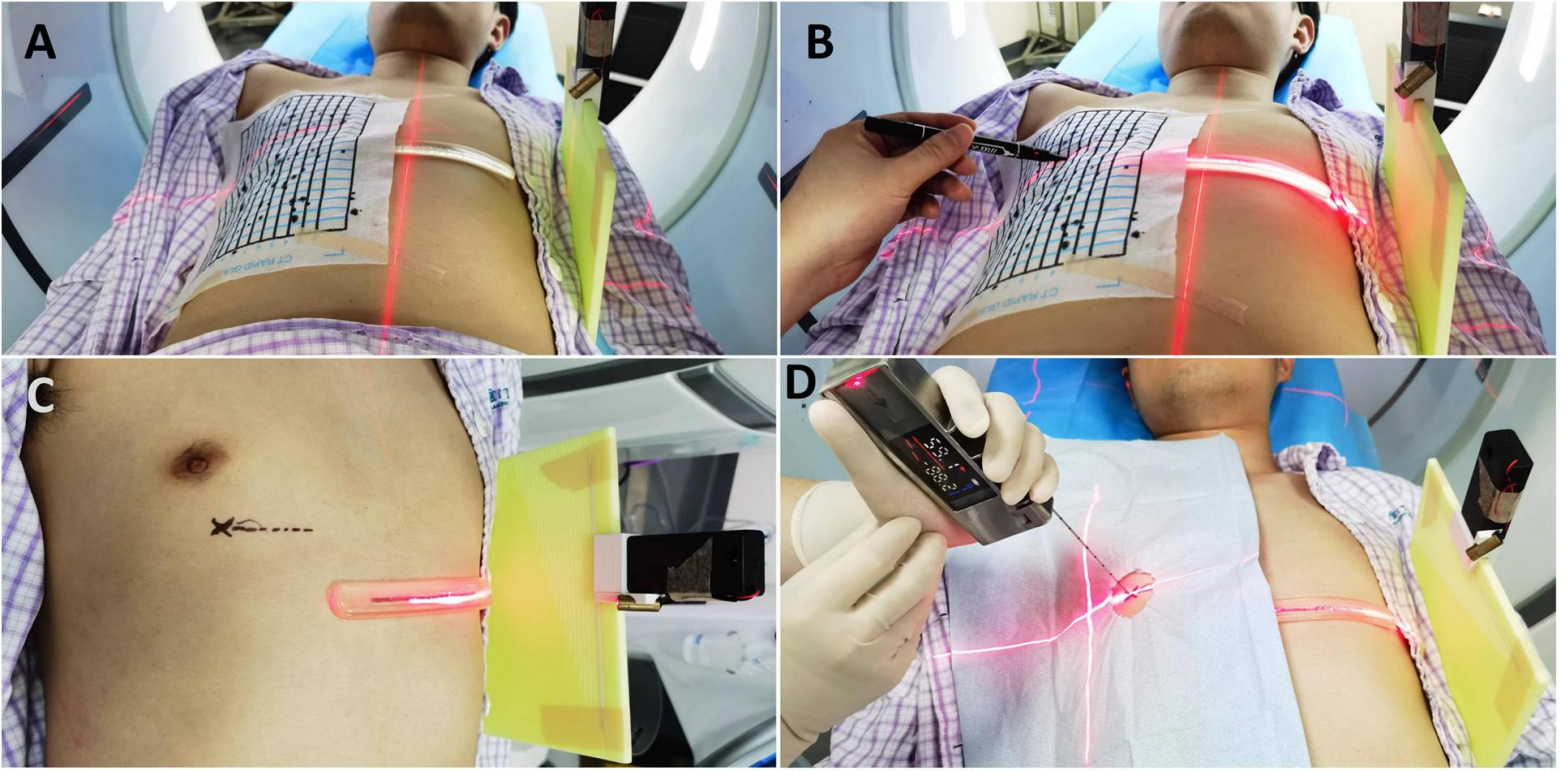
Percutaneous lung puncture by matching thoracic respiratory activity combined with laser angle guidance. **(A)** After the thoracic activity calibration plate and positioning paper are properly placed, the first ct scan is performed. **(B,C)** The patient is instructed to adjust the breathing amplitude to make the laser line coincide with the indicator band. At this time, the horizontal line corresponding to the puncture level and the intended puncture point are marked on the patient's skin according to the laser indication of the CT machine. **(D)** The patient is once again instructed to adjust the breathing amplitude to make the laser line projected by the positioning buckle of the thoracic activity calibration plate coincide with the indicator band. The laser angle guider is pushed and the puncture needle is advanced to the predetermined depth.

**Figure 4:**
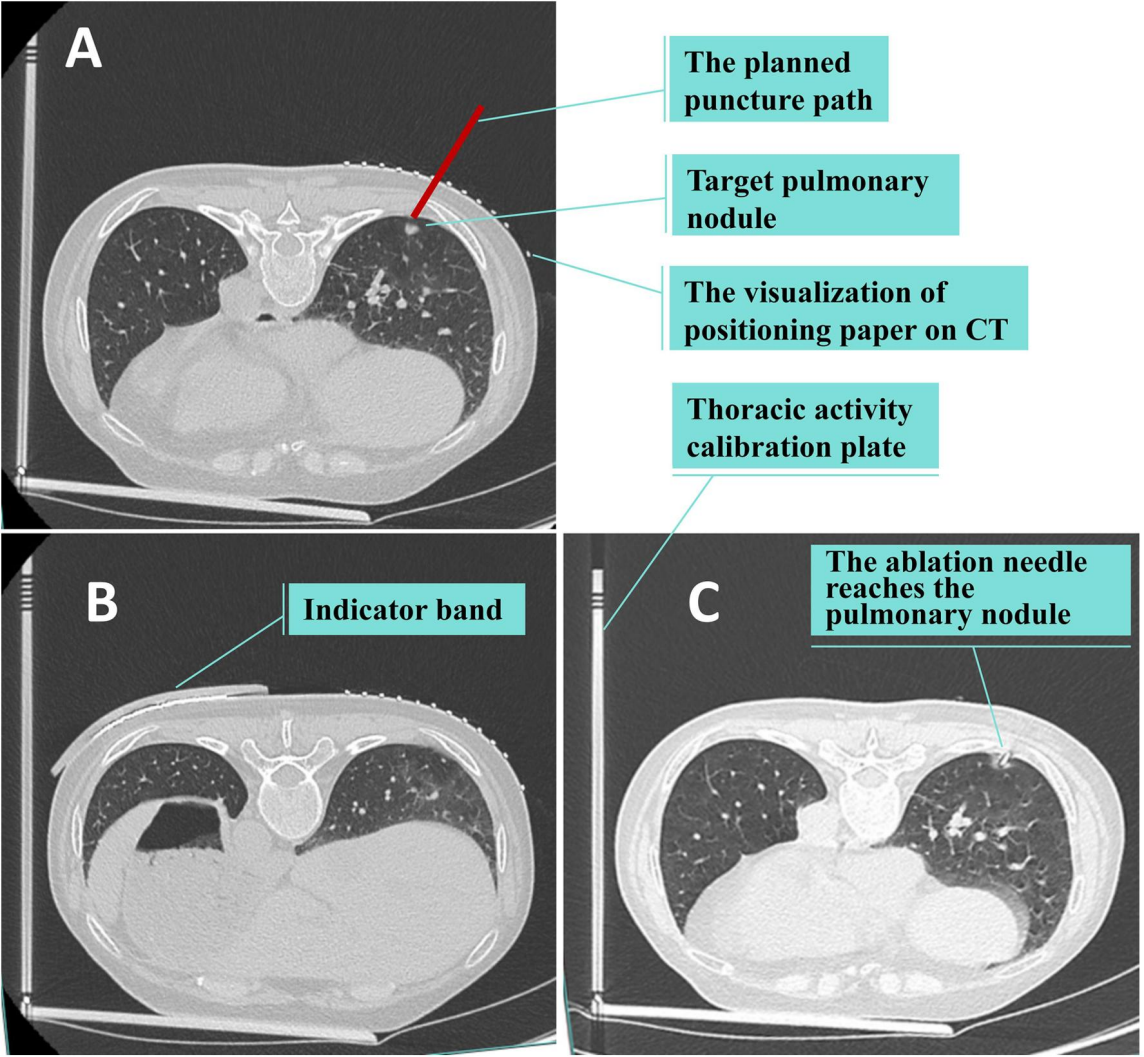
Ct scan. **(A,B)** The first CT scan is conducted to determine the puncture point, puncture level, and the scale of the thoracic activity calibration plate indicated by the attached indicator band on the chest skin. **(C)** The second CT scan is conducted again to determine whether the puncture needle has reached the predetermined position.

① Based on the location of the lung lesion, an appropriate body position is selected. The thoracic activity calibration plate is placed on the CT examination bed, with one side of it pressed between the chest and the bed surface, and the other side erected beside the thorax. Positioning paper (grid paper with lead wire) is affixed on the chest wall skin corresponding to the lung lesion, and the indicator band is attached on the side of the thorax with greater activity. The first CT scan is conducted to determine the puncture point, puncture level, and the scale of the thoracic activity calibration plate indicated by the attached indicator band on the chest skin (designated as “e”). The included angle between the intended needle insertion direction and the horizontal plane is measured (designated as “a” degrees), and the depth of the nodule from the skin is determined (designated as “b” centimeters). The positioning buckle is fastened at the “e” scale of the thoracic activity calibration plate, the laser is turned on, and the horizontal line corresponding to the “e” scale is projected. The patient is instructed to adjust the breathing amplitude to make the laser line coincide with the indicator band. At this time, the horizontal line (“c” line) corresponding to the puncture level and the intended puncture point are marked on the patient's skin according to the laser indication of the CT machine. ② The puncture needle is clamped on the laser angle guider and the laser on the guider is turned on. The guider is held and the puncture needle is inserted from the puncture point to beneath the skin. ③ The angle of the guider is adjusted to make the horizontal line of the laser coincide with the c line and to make the cross light of the laser parallel to the longitudinal and transverse axes of the CT bed respectively. ④ The angle of the guider is adjusted to make the X*Y* axis (parallel to the patient's horizontal plane) displayed on the laser angle meter be the intended needle insertion angle, and the Z axis (parallel to the patient's sagittal plane) angle be 0 degrees. ⑤ The patient is once again instructed to adjust the breathing amplitude to make the laser line projected by the positioning buckle of the thoracic activity calibration plate coincide with the indicator band. The laser angle guider is pushed and the puncture needle is advanced to the predetermined depth, then the guider and the puncture needle are separated. ⑥ A CT scan is conducted again to determine whether the puncture needle has reached the predetermined position. ⑦ If the position of the puncture needle is not perfect as seen on CT, the puncture is retreated as per the situation, and the needle is reinserted under the guidance of the LAG until the puncture needle reaches the perfect position. ⑧ Once the position of the puncture needle is satisfactory, operations such as biopsy, ablation, or release of the positioning needle are initiated. ⑨ After the operation, CT is rechecked to determine whether there are complications such as hemopneumothorax and pulmonary hematoma. If the lung compression due to pneumothorax exceeds 30%, or if the hemothorax is expected to have more than 300 ml of pleural effusion, closed thoracic drainage is performed under CT positioning. See Figures 3, 4.

#### Percutaneous lung puncture under the guidance of laser angle

**Figure 5:**
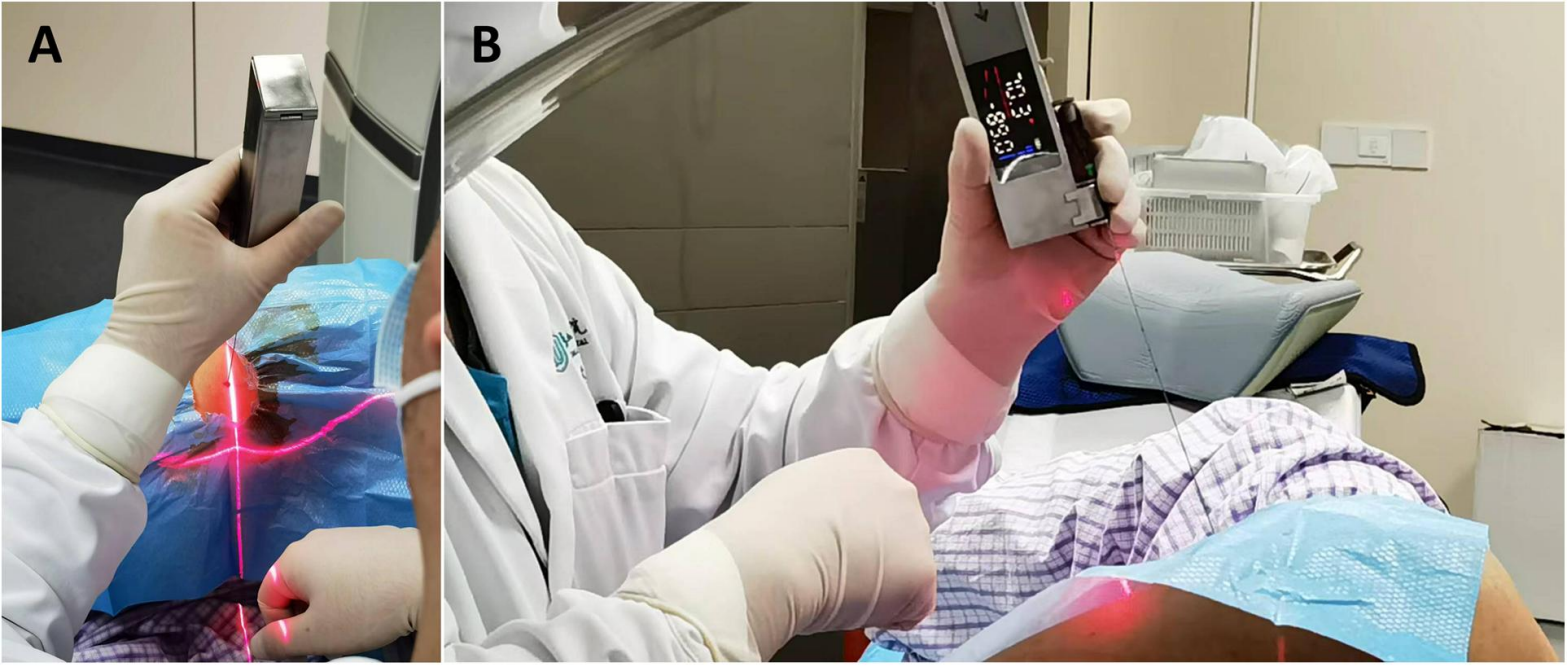
Perform the puncture under the guidance of the laser angle guider. The angle of the guider is adjusted to make the horizontal line of the laser coincide with the horizontal line corresponding to the puncture point and to make the cross light of the laser parallel to the longitudinal and transverse axes of the CT bed respectively.The angle of the guider is adjusted to make the X*Y* axis (parallel to the patient's horizontal plane) displayed on the laser angle meter be the intended needle insertion angle, and the Z axis (parallel to the patient's sagittal plane) angle be 0 degrees.

Except for not using the thoracic activity calibration plate, the other operation steps were the same as those of puncture under the technology of matching thoracic respiratory activity combined with laser angle guidance. See Figure 5.

#### Conventional percutaneous lung puncture

① Based on the location of the lung lesion, an appropriate body position is chosen, and positioning paper (grid paper with lead wire) is adhered to the chest wall skin corresponding to the lung lesion. The first CT scan is carried out to determine the puncture point and puncture level. The included angle between the intended needle insertion direction and the horizontal plane, as well as the depth from the nodule to the skin, are measured. ② The operator holds the puncture needle and determines the needle insertion angle based on his own experience and sensation, and punctures to the predetermined depth. ③ Another CT scan is conducted. If the needle insertion does not reach the satisfactory position, the puncture needle is withdrawn, the insertion direction is adjusted, and puncturing is repeated. CT scans and needle withdrawals and advancements are performed repeatedly until the puncture needle reaches the satisfactory position. ④ The subsequent operations are the same as those of puncture under the technology of matching thoracic respiratory activity combined with laser angle guidance.

### Observation indicators

The index data including the age, gender, operation projects (biopsy, ablation, location), size of pulmonary nodules, location of pulmonary nodules, distance from pulmonary nodules to the skin, rate of first puncture reached the predetermined position, number of CT scans, operation time, and incidence of complications of the patients in the three groups were collected.

The definitions of some of the observation indexes are as follows. ① Positioning: Percutaneous lung puncture and positioning needle positioning under CT guidance before lung nodule surgery. ② Ablation: Cryoablation of lung tumors through percutaneous lung puncture. ③ Distance from pulmonary nodules to the skin: The distance from the skin at the puncture point to the edge of the pulmonary nodules. ④.

Rate of first puncture reached the predetermined position: The ratio where the tip of the puncture needle has reached the predetermined position as shown by CT scan after the first insertion of the puncture needle. ⑤ Number of CT scans: The number of CT scans during the percutaneous lung puncture process. Represent the amount of radiation exposure that the patient receives during the puncture process. ⑥.

Operation time: The time from skin disinfection to the determination by CT scan that the tip of the puncture needle has reached the predetermined position.⑦ Complications: Complications such as hemothorax, pneumothorax, pulmonary hemorrhage, hemoptysis, air embolism, etc. caused by percutaneous lung puncture.

### Statistical analysis

*a priori* sample size was calculated using PASS (Version 2021). Based on preliminary data (first-attempt success rates: Con 24% vs. Ram 67%), with α = 0.05 and power = 0.9, the required minimum sample size was 264 patients. Our study met this requirement (*n* = 277). The measurement data following the normal distribution were expressed as mean ± standard deviation ($\bar{x}\pm s$), analysis of variance was used to analyze the differences among the three groups, and conduct pairwise comparisons by means of the LSD method. The categorical data were expressed as *n* (%), and analyzed with the chi-square test. If the expected frequency is <5, the Fisher exact test was used. A statistically significant level was predefined as α = 0.05. Statistical analysis was performed using *R* programming language (*R* Core Team, 2000).

## Results

### Comparison of the clinical features in 3 groups' patients

A total of 277 patients were included, among which there were 96 cases in the Con group, 93 cases in the Laser group, and 88 cases in the Ram group. There were no statistically significant differences in 6 clinical features including age, gender, operation projects, size of pulmonary nodules, position of pulmonary nodules, and distance from pulmonary nodules to skin among the three groups of patients (*P* > 0.05). See Table 1.

**Table 1:** Comparison of basic data among 3 groups of patients.

Observation indicator		Con group① (*n* = 96)	Laser group② (*n* = 93)	Ram group③ (*n* = 88)	F/Mean difference/*χ*^2^^b^value				*P* value			
					Intergroup	① vs. ②	② vs. ③	① vs. ③	Intergroup	① vs. ②	② vs. ③	① vs. ③
Operation projects, *n* (%)	biopsy	30 (31.25%)	33 (35.48%)	30 (34.09%)	0.840	0.569	0.489	0.234	0.933	0.752	0.783	0.889
	Location	59 (61.46%)	55 (59.17%)	51 (57.95%)								
	ablation	7 (7.29%)	5 (5.38%)	7 (7.95%)								
Age (years), Mean ± SD^a^, [95%CI]^c^		49.34 ± 14.23 [46.46, 52.23]	49.95 ± 14.45 [46.97, 56.92]	51.02 ± 13.27 [48.21, 53.83]	0.336	−0.602	−1.076	−1.679	0.715	0.988	0.937	0.792
Gender, *n* (%)	Male	49 (51.04%)	53 (56.99%)	46 (52.27%)	0.741	0.673	0.406	0.028	0.690	0.412	0.524	0.867
	Female	47 (48.96%)	40 (43.01%)	42 (47.73%)								
Size of pulmonary nodules (mm), Mean ± SD, [95% CI]		12.17 ± 3.70 [11.42, 12.92]	11.94 ± 3.44 [11.23, 12.64]	11.91 ± 3.73 [11.12, 12.70]	0.143	0.231	0.026	0.258	0.867	0.959	1.000	0.953
Position of pulmonary nodules, *n* (%)	Right Upper Lobe	18 (18.75%)	12 (12.90%)	16 (18.18%)	5.70	4.560	1.790	2.499	0.681	0.336	0.774	0.645
	Right Middle Lobe	19 (19.79%)	14 (15.05%)	15 (17.05%)								
	Right Lower Lobe	17 (17.71%)	19 (20.43%)	13 (14.77%)								
	Left upper lobe	18 (18.75%)	28 (30.11%)	25 (28.41%)								
	Left lower lobe	24 (25.00%)	20 (21.51%)	19 (21.59%)								
Distance from the pulmonary nodule to skin (mm), Mean ± SD, [95% CI]		51.07 ± 10.18 [49.01, 53.13]	53.20 ± 10.22 [51.10, 55.31]	50.47 ± 10.63 [48.21, 52.72]	1.775	−2.131	2.738	0.607	0.171	0.390	0.219	0.971

^a^ SD, standard deviation; ^b^χ^2^, Chi-square test; ^c^95% CI, 95% Confidence Interval.

### Comparison of indicators related to puncture techniques

The rate of first puncture reached the predetermined position in the Ram group was higher than that in the Laser group and higher than that in the Con group, which were 67.05% > 37.63% > 23.96% in sequence, intergroup *P* < 0.001. The number of CT scans during the puncture process in the Ram group was less than that in the Laser group and less than that in the Con group, which were 3.66 ± 1.06 times < 4.09 ± 1.05 times < 4.50 ± 1.08 times in sequence, intergroup *P* < 0.001. The operation time in the Ram group was less than that in the Laser group and less than that in the Con group, which were 23.05 ± 13.89 min <28.83 ± 13.78 min <35.14 ± 14.20 min in sequence, intergroup *P* < 0.001. The overall complication rate in the Ram group was lower than that in the Laser group and lower than that in the Con group, with the rates being 7.95% < 16.13% < 26.04% in sequence. However, there was no statistically significant difference (intergroup *P* = 0.146). Further analysis of complication subtypes revealed that only the incidence of pneumothorax showed a statistically significant difference among the three groups. The incidence rates in the Ram group, Laser group, and Con group were 19.79% > 12.90% > 5.68% respectively, intergroup *P* = 0.018. See Table 2.

**Table 2:** Comparison of intra—operative and post—operative observation indicators among 3 groups of patients.

Observation indicato		Con group① (*n* = 96)	Laser group② (*n* = 93)	Ram group③ (*n* = 88)	F/Mean difference/χ^2^^b^value				*P* value			
					Intergroup	① vs. ②	② vs. ③	① vs. ③	Intergroup	① vs. ②	② vs. ③	① vs. ③
First puncture reached the predetermined position, *n* (%), [95% CI^c^]		23 (23.96%) [16.53%, 33.39%]	35 (37.63%) [28.45%, 47.79%]	59 (67.05%) [56.68%, 75.97%]	36.153	4.154	15.668	34.503	*P* < 0.001	0.042	*P* < 0.001	*P* < 0.001
Number of CT scans (times), Mean ± SD^a^, [95% CI]		4.50 ± 1.08 [3.96, 4.42]	4.09 ± 1.05 [3.77, 4.27]	3.66 ± 1.06 [3.40, 3.90]	14.394	0.414	0.427	0.841	*P* < 0.001	0.024	0.021	*P* < 0.001
Operation time (min), Mean ± SD, [95% CI]		35.14 ± 14.20 [31.26, 37.01]	28.83 ± 13.78 [25.99, 31.67]	23.05 ± 13.89 [20.10, 25.99]	14.489	5.307	5.783	11.090	*P* < 0.001	0.009	0.006	*P* < 0.001
Complications, *n* (%), [95%CI]		25 (26.04%) [18.31%, 35.62%]	15 (16.13%) [9.78%, 25.46%]	7 (7.95%) [3.59%, 16.22%]	12.118	3.196	3.804	11.498	0.146	0.526	0.433	0.021
	Hemothorax	1 (1.04%) [0.00%, 6.95%]	1 (1.08%) [0.00%, 7.12%]	1 (1.14%) [0.00%, 7.33%]	0.004	0.001	0.002	0.004	0.998	0.982	0.969	0.951
	pneumothorax	19 (19.79%) [13.27%, 28.80%]	12 (12.90%) [7.58%, 21.64%]	5 (5.68%) [1.96%, 12.31%]	8.085	1.635	2.771	8.059	0.018	0.201	0.096	0.005
	pulmonary hemorrhage	2 (2.08%) [0.57%, 7.28%]	1 (1.08%) [0.19%, 5.85%]	0 (0%) [0.00%, 4.18%]	2.623	0.314	1.337	2.622	0.269	0.575	0.248	0.105
	hemoptysis	3 (3.13%) [1.07%, 8.79%]	1 (1.08%) [0.19%, 5.84%]	1 (1.14%) [0.20%, 6.16%]	1.358	1.004	0.002	0.889	0.507	3.160	0.969	0.343

^a^ SD, standard deviation; ^b^χ², chi-square test; ^c^95% CI, 95% confidence interval.

## Discussion

**Figure 6:**
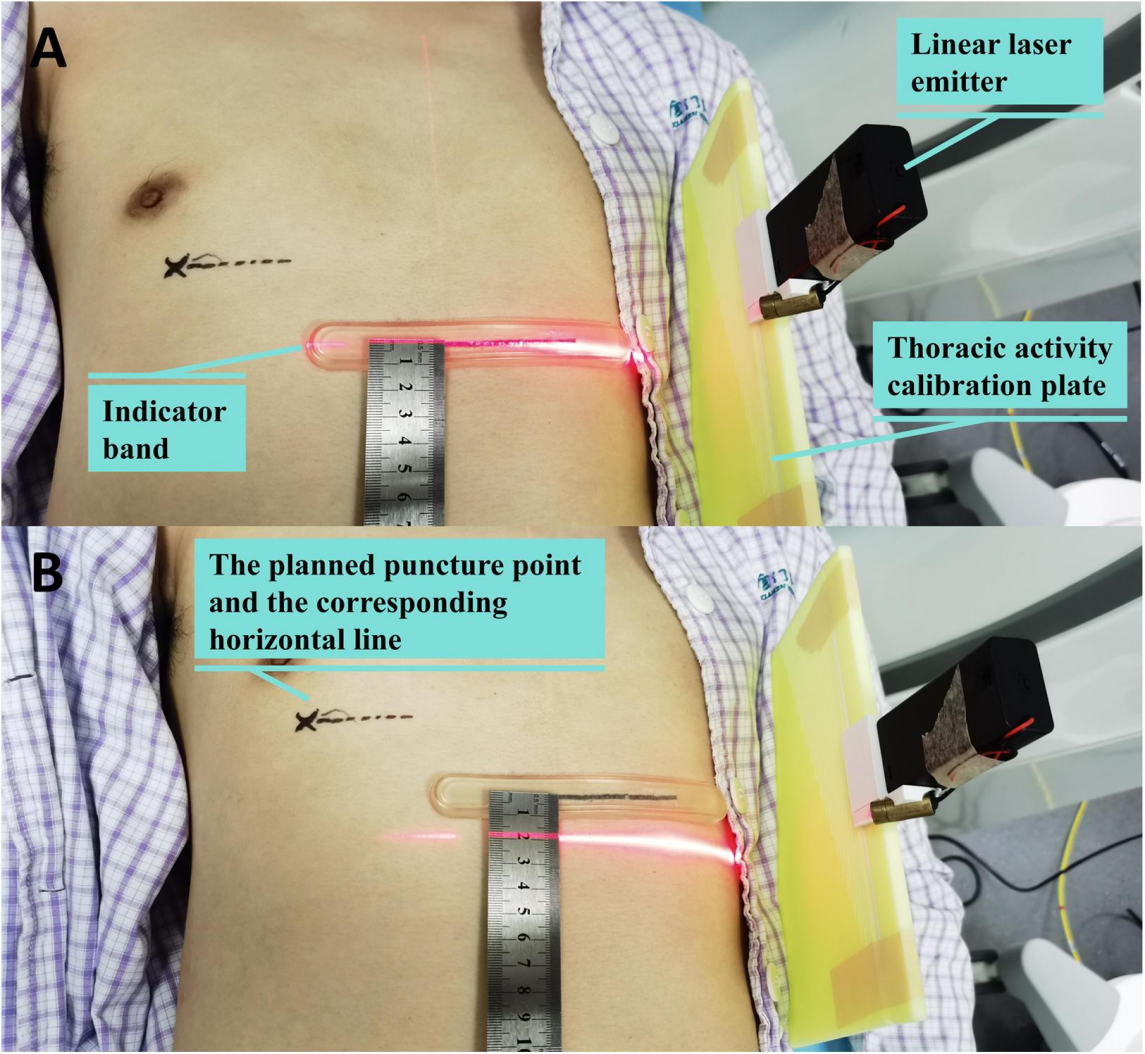
The up-and-down movement range of the thorax reaches 2 cm when deep breath. **(A)** The laser beam shines on the indicator band when exhaling deeply; **(B)** The laser beam shines 2 cm below the indicator band when inhaling deeply.

When performing percutaneous lung puncture, accurately pushing the puncture needle to the target location is crucial for the effectiveness of biopsy, ablation and positioning. During the operation of CT-guided percutaneous lung puncture, factors such as the patient changing position due to pain or other causes, the contraction of chest wall muscles caused by pain, the respiratory movement of the lungs, and the tremor of the operator's hand, all have a significant impact on the accuracy of the puncture (17, 18). The relative position between the lung lesion and the body surface changes continuously during the breathing process. As patients cannot precisely control the breathing amplitude, the corresponding body surface positions of the lung lesion at the time of CT scanning, marking the puncture point, and conducting the puncture are all dissimilar, and the puncture accuracy is significantly compromised, naturally. A minor deviation can lead to a substantial error. Among them, thoracic respiratory movement and the precise direction of needle insertion are even more significant factors influencing the puncture accuracy. See Figure 6.

Given that the movement of the lungs and that of the thorax during respiratory activities are interlinked, during smooth breathing, at the same spatial position of the thorax, the corresponding spatial position of the lungs is largely the same. Our team designed a thorax activity calibration plate. By matching the state of thorax activity, the patient's thorax can be in the same position during CT scanning, determining the puncture point, and pushing the puncture needle. meaning that the lungs are approximately at the same position as well. Thus, the influence of respiratory movement on the puncture is overcome. Furthermore, we have also designed a laser angle guide. Through angle display and laser indication, it can guide the pushing direction of the puncture needle in three-dimensional space, enabling the puncture needle to push precisely in the preset direction.

It can be seen from the results of this study that, compared with conventional puncture, percutaneous lung puncture under the guidance of laser angle significantly improves the accuracy of puncture, reduces the number of CT scans and operation time during the puncture process, and at the same time, the incidence of complications such as hemopneumothorax and hemoptysis also significantly decreases. On this basis, percutaneous lung puncture combined with matching thorax respiratory activity and laser angle guidance further improves the accuracy of puncture. When puncturing lung lesions smaller than 2 cm, 61.61% of patients can reach the predetermined position with one needle insertion.

Regarding studies on overcoming the influence of respiratory movement on percutaneous lung puncture, apart from respiratory gating technology (11, 19) and magnetic navigation technology (20, 21), there are no other public reports at present. The thorax activity calibration plate we designed has addressed this issue to a certain extent. Currently, the commercially available percutaneous lung puncture assisting devices are similar to our research in terms of reducing the frequency of adjusting the puncture position and CT scans during the puncture process. Bodard S (22) reported that the average number of needle adjustment times for machine assisted puncture and conventional puncture were 2.7 ± 2.6 times vs. 6 ± 4 times. Lanouzière MB (23) reported that the average number of CT scans for CT Navigation™ magnetic navigation machine assisted puncture and conventional puncture were 8 (Interquartile Range: 7–10) times vs. 9 (Interquartile Range: 8–11) times respectively. The complication rate is also similar to that of our study. Previous similar studies mainly centered on the success rate of pathological biopsy (24, 25), whereas we place greater emphasis on the precise targeting of the puncture needle. Given the numerous differences in the observed indicators, it is challenging to directly compare the superiority of the robot assisted puncture device and the assisting device we designed. However compared with expensive advanced equipment such as respiratory gating devices and magnetic navigation devices, the advantage of the thorax activity calibration plate lies in its straightforward production and low cost. All the components can be fabricated using a basic 3D printer, and it is also easy to operate, which makes it applicable in resource-limited environments and facilitating its popularization in primary hospitals.

It is necessary to point out that there are some deficiencies in this study. First of all, this is a single-center retrospective study, inevitably having some inevitable biases. Additionally, the techniques of percutaneous lung puncture by different doctors differ, and it is unclear whether the use of the thorax activity calibration plate and the laser angle guide for assisting puncture is beneficial for every doctor. Hence, the conclusion of this study still requires confirmation through multi-center studies. Secondly, the implementation of percutaneous lung puncture combined with matching thorax respiratory activity and laser angle guidance is based on the application of the two tools, namely the thorax activity calibration plate and the laser angle guide. However, these two tools still have considerable room for improvement and a long way to go before commercial promotion. Certainly, interested colleagues can fabricate them on their own based on the introduction in this article and utilize them for scientific research.

## Conclusions

In conclusion, puncture under the guidance of matching thorax respiratory activity and laser angle is a new idea. This technique demonstrates promising results and may serve as a low-cost adjunct in CT-guided lung procedures, warranting further evaluation in larger, prospective studies.

## Data Availability

The raw data supporting the conclusions of this article will be made available by the authors, without undue reservation.
